# The Role of Autochthonous *Levilactobacillus brevis* B1 Starter Culture in Improving the Technological and Nutritional Quality of Cow’s Milk Acid-Rennet Cheeses—Industrial Model Study

**DOI:** 10.3390/foods13030392

**Published:** 2024-01-25

**Authors:** Barbara Sionek, Anna Okoń, Anna Łepecka, Dorota Zielińska, Danuta Jaworska, Katarzyna Kajak-Siemaszko, Katarzyna Neffe-Skocińska, Monika Trząskowska, Marcelina Karbowiak, Piotr Szymański, Zbigniew J. Dolatowski, Danuta Kołożyn-Krajewska

**Affiliations:** 1 Department of Food Gastronomy and Food Hygiene, Institute of Human Nutrition Sciences, Warsaw University of Life Sciences (SGGW), 02-776 Warsaw, Poland; dorota_zielinska@sggw.edu.pl (D.Z.); danuta_jaworska@sggw.edu.pl (D.J.); katarzyna_kajak_siemaszko@sggw.edu.pl (K.K.-S.); katarzyna_neffe_skocinska@sggw.edu.pl (K.N.-S.); monika_trzaskowska@sggw.edu.pl (M.T.); marcelina_karbowiak@sggw.edu.pl (M.K.); danuta_kolozyn_krajewska@sggw.edu.pl (D.K.-K.); 2 Department of Meat and Fat Technology, Prof. Waclaw Dabrowski Institute of Agricultural and Food Biotechnology—State Research Institute, 36 Rakowiecka St, 02-532 Warsaw, Poland; anna.okon@ibprs.pl (A.O.); anna.lepecka@ibprs.pl (A.Ł.); piotr.szymanski@ibprs.pl (P.S.); zbigniew.dolatowski@ibprs.pl (Z.J.D.)

**Keywords:** cheese, probiotics, *Lactobacillus*, starter culture, raw milk, dairy products

## Abstract

In the study, an attempt was made to develop an innovative technology for cheese manufacturing. It was hypothesized that selected autochthonous lactic acid bacteria as a starter culture are more suitable for the production of acid-rennet cheeses of good technological and sensory quality. The study aimed to assess the possibility of using the strain *Levilactobacillus brevis* B1 (*L. brevis* B1) as a starter culture to produce acid-rennet cheeses using raw cow’s milk. Two variants of cheese were manufactured. The control variant (R) was coagulated with microbial rennet and buttermilk, and the other variant (B1) was inoculated with rennet and *L. brevis* B1 starter culture. The effect of the addition of these autochthonous lactic acid bacteria on selected physicochemical characteristics, durability, the composition of fatty acids, cholesterol, Iipid Quality Indices, and microbiological and sensory quality of acid-rennet cheeses was determined during a 3-month period of storage. The dominant fatty acids observed in the tested cheeses were saturated fatty acids (SFA) (68.43–69.70%) and monounsaturated fatty acids (MUFA) (25.85–26.55%). Significantly higher polyunsaturated fatty acid (PUFA) content during storage was observed for B1 cheeses. The B1 cheeses were characterized by lower cholesterol content compared to cheese R and showed better indexes, including the Index of atherogenicity, Index of thrombogenicity, DFA, OFA, H/H, and HPI indexes, than the R cheese. No effect of the tested *L. brevis* B1 on sensory quality was observed in relation to the control cheeses during 3 months of storage. The results of the research indicate the possibility of using the *L. brevis* B1 strain for the production of high-quality, potentially probiotic acid-rennet cheeses.

## 1. Introduction

Cheese is a nutrient-dense dairy food, providing protein, fats, B vitamins, fat-soluble vitamins, and minerals including calcium. The high nutritional attractiveness of cheeses is primarily due to the content of wholesome animal protein, which is characterized by a very high digestibility and a low content of lactose in some types, so that is appropriate for people with lactose intolerance. 

From the dietetic point of view, it was found that although consumption of whole milk is associated with a higher risk of developing cardiovascular disease (CVD) [1,2] data from the meta-analysis indicated a nonlinear inverse association between cheese consumption and the risk of CVD [3]. Moreover, intake of yogurt, low-fat milk, butter, and acid cheese is associated with lower or no risk of the development of cardiovascular diseases, type 2 diabetes, and metabolic syndrome [1].

The acid-rennet cheeses are classified as high-quality dairy products. Available in low-fat and nonfat forms, constitutes a common type of cheese in Anglo-Saxon and Central-Eastern European countries [4]. The widespread popularity of acid-rennet cheeses results from the multi-generational tradition of their consumption, established eating patterns, their large availability in a rich assortment, and their affordable retail price. Not without significance is the public’s perception of cottage cheese as a healthy food [4]. Different cheeses are characterized by a unique flavor that depends on the presence and interplay of various odor-active compounds, including heptanal, hexanal, styrene, acetoin, acetic acid, furfural, butanoic acid, limonene, butyl acetate, and many more [5]. Some recent innovations and developments in the acid-rennet cheesemaking process include the addition of probiotic cultures and prebiotics and the extension of shelf-life through the incorporation of bacteriocin-producing cultures [6].

It is suggested that perhaps specific nutrients, like calcium and conjugated linoleic acid (CLA) in cheese, may act protectively for the cardiovascular system. The distinctive composition of fatty acids and incidence of autochthonous bacteria, as well as a starter culture, are also influential [7,8].

Starter cultures consist of bacteria, yeasts, molds, and their combination, among which lactic acid bacteria (LAB) and yeasts are the most extensively used microorganisms. The use of starter cultures is a common practice in the food industry worldwide. Corbo et al. pointed out that the main reason manufacturers use commercial starters is fear of product failure [9]. For that reason, the commercialization of several products, such as bioprotective cultures, starters, or probiotics, is taking place to provide foods with unique sensory and nutritional values, along with potential health benefits, as well as to ensure food safety. Nowadays, commercial starter cultures have mostly replaced traditional ones for industrial-scale production because of their defined strain identification, high reproducible performance, and high phage resistance [10]. Nevertheless, commercial starters are not suited to manufacture traditional and unique dairy products due to the possibility of dominating the autochthonous milk microbiota and causing undesirable standardization of the final product [11]. The fermentation conducted by various strains of LAB (i.e., *L*. *fermentum* NB02, *L*. *johnsonii* LA1, *L*. *reuteri* SD2112, and *L*. *acidophilus* LA-5) and the secretion of bacterial esterases can contribute to increasing the content of bioactive compounds like free phenolic acids and flavonoids, which enhance antioxidant activity [12]. In the study of Zhang et al. (2023), was shown that bioactive compounds could stimulate the proliferation of beneficial human gut microbiota [13]. The recent literature indicates that there is a strong necessity for the isolation of new bacterial strains from the wild that can be used as starter cultures to enhance the genetic diversity of the LAB collection designed for both traditional and innovative dairy applications [11].

In recent years, consumer interest in dairy products has been growing, including artisanal cheeses produced using traditional methods. For example, cheeses made from raw milk are characterized by more intense flavor and aroma compared with cheeses made from pasteurized milk [14,15]. To preserve the traditional character of these products, they are produced from raw milk with natural microbiota [16].

Therefore, this study aimed to assess the possibility of using the selected autochthonous LAB as a starter culture for the manufacturing of high-quality acid-rennet cheeses from cow’s milk. A novelty of the conducted study was the application under industrial conditions of a unique strain of lactic acid bacteria—*Levilactobacillus brevis* B1, isolated from Bundz, mountain sheep milk cheese traditionally produced in the Podhale region (Poland). The research included the effect of *Levilactobacillus brevis* B1 addition on selected physicochemical characteristics, storage time, composition of fatty acids, cholesterol, Lipid Quality Indices, and microbiological and sensory quality of acid-rennet cheeses. 

## 2. Materials and Methods

### 2.1. Materials

#### 2.1.1. *Levilactobacillus brevis* B1 Starter Culture Bacteria

The autochthonous *L. brevis* B1 strain has been used as a starter culture. The *L. brevis* B1 strain came from the collection of the Department of Food Gastronomy and Food Hygiene, Institute of Human Nutrition Sciences, University of Life Sciences, and was isolated from Polish regional cheese “Bundz” made from sheep’s milk. The *L. brevis* B1 strain was selected in a previous study, in which its effect on various quality characteristics, sensory, and shelf life of food of animal origin was evaluated [17]. The *L. brevis* B1 strain can survive in conditions that simulate the human digestive tract and can colonize the intestinal epithelium. The strain shows selected probiotic properties and exhibits antimicrobial potential against *Listeria monocytogenes*, *Bacillus subtilis*, *E. coli*, *Enterococcus feacium*, and *Salmonella enteritidis.* The strain and its properties are protected (Patent No.: P.426002/ UP RP).

#### 2.1.2. Starter Culture Preparation

The *L. brevis* B1 strain was kept at −80 °C in MRS broth (Merck, Darmstadt, Germany) with 20% glycerol added. Preparation of the *L. brevis* B1 strain consisted of activating frozen bacteria, suspending them in MRS broth (LabM, UK), and incubating them at 37 °C for 24 h. The bacteria cells were washed in PBS (phosphate-buffered saline), and then the milk was inoculated with them. The inoculum was 1% (*v*/*v*) and the initial count of bacteria was approx. 8.0 log CFU mL^−1^.

#### 2.1.3. Acid-Rennet Cheeses Manufacture

Cheese production took place in a small production plant located in central Poland. The plant specializes in the processing of raw milk. Two variants of acid-rennet cheeses were manufactured. The first, control sample (R), was coagulated with microbial rennet and buttermilk, and the other variant of cheese was inoculated with rennet and *L. brevis* B1 strain (B1). The buttermilk used in the study was a fermented by-product of butter production. It was created by separating fat from cream. The manufacturer followed the procedure: the milk was gently heat treated to 36 °C, and calcium chloride (0.001%) was added to obtain the correct curd and rennet functions. Buttermilk (the LAB concentration was approx. 8 log CFU mL^−1^) or starter culture: *L. brevis* B1 strain was added to the milk. Then microbial rennet (0.005%) was added to the mixture. The milk was allowed to form a clot for 1 h at 36 °C. Then the clot was cut (36 °C, 20 min). The curd was mechanical-thermal treated (i.e., grinding, rinsing, and drying the grain, taking away excess whey). The cheeses were formed, pressed, and salted. The cheeses were placed in a ripening room and matured at a temperature of 8 °C, and relative humidity was controlled at 90% for 7 days. Samples for analysis were taken after production (0 days) and after 1, 2, and 3 months of refrigerating (4 °C ± 2 °C) storage.

### 2.2. Methods

#### 2.2.1. Microbiological Analyses

Microbiological analysis of acid-rennet cheeses was performed for the detection of *Listeria monocytogenes* according to the ISO 11290-1:2017 procedure [18]; ALOA agar (Bio-Rad, Hercules, CA, USA) and PALCAM agar (LabM, USA) were used, *Salmonella* spp. (ISO 6579-1:2017-04) [19] on the RAPID agar (Bio-Rad, Hercules, CA, USA). To enumerate *Enterobacteriaceae* (ENT), MacConkey agar (LabM, UK) was used according to de Boer (1998) [20]. The number of LABs was determined by the pour-plate method on an MRS agar (Biokar Diagnostics, Allonne, France) according to ISO 15214:1998 [21]. The number of yeasts and molds was performed on YGC agar (Sabouraud Dextrose with Chloramphenicol LAB-Agar, Biomaxima, Lublin, Poland) according to ISO 21527-1:2008 and ISO 21527-2:2008 [22,23]. The number of microorganisms was expressed as log CFU g^−1^. The count of bacterial cells was evaluated immediately after production and after 1, 2, and 3 months of refrigerated storage (4 °C ± 2 °C).

#### 2.2.2. Chemical Composition

The water content was measured using the weighing method according to ISO standards (ISO 1442:1997) [24], protein content according to the method PN-A-04018:1975/Az3:2002 [25], the content of free fat according to the method ISO 8262-3:2005 [26], chloride content according to the method ISO 1841-2:1996 [27], andphosphorus according to the method PN-A-82060:1999 [28].

#### 2.2.3. Physicochemical Parameter Analysis

Titratable acidity, pH, and oxidation-reduction potential (ORP) of cheese were determined according to the method described by Łepecka et al. (2022) [29].

Water activity (a_w_) was determined with the Pawkit Water Activity Meter, USA. Approximately 5 g of cheese was placed in a measuring cup in the device. 

The instrumental color of the samples was quantitatively determined using a Minolta CR-300 spectrophotometer (Konica Minolta, Tokyo, Japan). During the measurements, a standard CIE observer was used: 2°, illuminant D65, and diaphragm diameter 8 mm. The calibration was conducted with the white tile standard (L* 99.18; a*, 0.07; b*, 0.05).

#### 2.2.4. Texture Profile Analysis (TPA)

TPA was analyzed using the CT3 Texture Analyzer (Brookfield Ametek, Middleborough, MA, USA). The samples were compressed twice to 50% of their original height at a speed of 0.50 mm s^−1^ with a cylindrical head (38.1 mm in diameter, 20 mm high) at a maximum pressing force of 10.000 g. Each sample was cut into a 20 mm square. The following parameters were determined: hardness 1 (maximum force of the first compression (N)); hardness 2 (maximum force of the second compression (N)); adhesiveness (the work necessary to overcome the attractive forces between the food surface and other materials it contacts (mJ)); cohesiveness (the extent to which the material can be deformed before fracture (dimensional); springiness (the speed at which the deformed material returns to its undistorted state after the deforming force removal (mm)); gumminess (energy needed to break up a semi-solid food product that is ready-to-swallow, i.e., a product with a low degree of hardness and a high degree of cohesiveness (N)); chewiness (energy required to chew solid food until it is ready to swallow, i.e., a product of hardness, cohesiveness, and elasticity (mJ)); and average peak load. All the measurements were replicated three times.

#### 2.2.5. The Fatty Acid, Cholesterol, and Lipid Quality Indices Measurement

The fatty acid content was determined by gas chromatography with a flame ionization detector, according to Łepecka et al. (2022) [29]. The results are presented as the sum of saturated, monounsaturated, polyunsaturated, trans, n-3, and n-6 fatty acids (%).

The cholesterol content was determined by lipid fraction extraction, fatty acid esterification, and cholesterol derivatization in the presence of the standard. The sample was analyzed by gas chromatography with flame ionization detection (GC-FID). The value was expressed in mg/100 g of product.

Lipid Quality Indices: The indices: AI—Index of atherogenicity, TI—Index of thrombogenicity, DFA—Hypocholesterolemic fatty acids, OFA—Hypercholesterolemic fatty acids, H/H—The ratio of hypocholesterolemic and hypercholesterolemic fatty acids; HPI—Health Promoting Index were calculated according to Chen and Liu (2020) [30] and Paszczyk and Łuczyńska (2020) [31].

#### 2.2.6. Sensory Evaluation

The quantitative descriptive analysis (QDA) was used for the sensory assessment (ISO 13299:2016-05 standard) [32]. A linear scale (100 mm) converted to numerical values (0–10 c. u.) was used. The set of attributes was chosen and defined during a panel discussion and then verified in a preliminary test. Before proper assessment, a set of cheese samples was presented to the panelists. The following attributes were established for the evaluation of studied samples: 5 attributes of odor (of milk, sour, creamy, irritating, other), 1 attribute of color (color intensity, where: 0-white, 10-yellow), 3 texture attributes (softness, moisture, and elasticity), 5 flavor attributes (milk’s fermentation, cow milk’s, fatty, off, other cheese). Based on all the above-mentioned attribute characteristics, a sensory-trained panel assessed an overall sensory quality (low-very high) for each sample. The anchor marks of the tested attributes ranged from low (the left side) to very high intensity (the right side of the scale).

The cheese samples were cut into portions (average piece size: about 15 g) and placed in odorless, disposable boxes, covered with lids. The boxes were individually coded with 3-digit codes and given in a random order for the evaluation. 

The trained 8-member panel (7 women and 1 man, aged 28–58) was extensively and formally tested before being selected, according to the ISO standard (ISO 8586:2012) [33]. The panelists possessed 4 to 18 years of both theoretical and practical experience in sensory procedures and evaluation of different food products with various methods. The assessor’s ability to differentiate product samples by various concentrations of volatile and non-volatile stimuli was verified. 

The evaluators were separated from each other to conduct the assessment in full focus. The walls of the room, as well as all of the tables, were white. The ambient temperature was 22–23 °C. The assessment and condition mode were determined following Meilgaard et al. (2006) [34].

#### 2.2.7. Statistical Analyses

The experiments were conducted in two independent experimental series (batches) of the product, which were manufactured under industrial conditions. Three randomly selected cheese samples from each batch were analyzed. Therefore, a total of 6 individual results were analyzed, with 3 individual results from each batch of cheese, except for the color analysis, where 5 measurements were made for each cheese. The tests were performed at specific time intervals (0, 1, 2, and 3 months). All data were expressed as mean values ± standard deviations. Statistical analysis was performed using one-way analysis of variance (ANOVA), followed by Tukey’s test. The significance of the calculated coefficients was established at the *p* < 0.05 level. The obtained data were developed using the statistica program version 13.1 software (TIBCO Software Inc., Palo Alto, CA, USA).

## 3. Results and Discussion

### 3.1. Chemical Composition of Acid-Rennet Cheeses

Due to the fat content, both variants of acid-rennet cheeses can be classified as medium-fat, but based on the water content, they belong to semi-hard cheeses. The content of NaCl was typical for these types of cheeses (Table 1). A significantly lower content of water was found in the variant of cheese R compared to cheese B1 (*p* < 0.05). The protein content in R cheese was significantly higher than in the B1 cheese variant (*p* < 0.05). Significantly lower fat content was observed for cheese B1 compared to cheese R (*p* < 0.05). It is concordant with the results of other researchers who reported that the decrease in cheese water content is related to the increase in protein and fat content [35,36]. Similar salt and phosphorus contents were found among the studied cheeses. Sodium chloride, in addition to having a direct impact on the perception of the salty taste of cheese, also indirectly contributes to the formation of flavor as a result of the action of microorganisms, enzymatic activity, lactose metabolism, and changes in acidity [37].

### 3.2. Physicochemical Analysis

Table 2 shows average values of pH, titratable acidity, oxidation-reduction potential, water activity, and color of tested cheeses after production and during storage. The water activity of cheeses after production was on average 0.995 in the B1 sample, while in the R sample, it was slightly lower, i.e., 0.975. The water activity of cheeses depends on the technological process, ripening time, and the species of animal from which the milk comes [38,39,40]. The value of water activity after 3 months of cheese storage decreased to 0.960 and 0.947 in the B1 and R samples, respectively; however, they were still at a high level, which is characteristic of this type of product.

After production, there was no significant difference in pH between the cheese varieties, but it differed significantly during storage. The data presented in Table 2 highlights a significant impact of B1 culture on the pH levels of the products. In all of the samples, a decline in pH was noted after 1 and 2 months of storage, followed by an increase in pH after 3 months of storage to the value of 5.02–5.19. The decrease in pH after a month of storage was probably caused by the fermentation of lactose to lactic acid. At the end of storage, the increase in cheese pH was likely associated with the proteolytic process, which releases large amounts of nitrogenous alkali compounds [35,41]. Regarding the titratable acidity, the data obtained showed values ranging from 88.00 to 225.50 °SH (cheese B1) and from 79.15 to 229.25 °SH (cheese R) during 3 months of storage. In particular, during the first month of storage, all samples showed a decrease in titratable acidity compared to the data observed after production. A significant increase in this parameter was observed at 3 months, possibly due to lactic acid formation from residual lactose still present in the cheese [41,42].

At the beginning, the R variant exhibited a higher ORP value (479.65 mV), and the lower was for the B1 cheese (398.05 mV, *p* < 0.05) (Table 2). After 1 month of storage, a significant ORP decrease (*p* < 0.05) was noted only for the R treatment (392.83 mV), followed by an increase after 2 months of storage to 519.48 mV (*p*> 0.05). These changes might be the result of the enzymatic and microbial activity of the cheese microbiota [29,43]. Cheese is a complex system of pro- and anti-oxidant components; the decrease in antioxidant activity mainly depends on the intensity of oxidative reactions and the degradation of antioxidant components such as vitamins and enzymes, while the increase in cheese can be related to the formation of peptides during ripening, which has antioxidant properties [44,45]. Supplementation with natural antioxidants, selenium, and vitamin E significantly improved the antioxidant capacity of fresh cheeses, inhibiting lipid oxidation and improving oxidative stability during ripening [46,47]. According to some authors, the high content of fat in cheese is a factor predisposing it to lipid oxidation and higher redox potential [29,48]. 

Acid-rennet cheese with buttermilk (R) did not differ significantly in brightness immediately after production and 1 month (L* = 85.03 and 84.22, respectively) as compared with acid-rennet cheese with *L. brevis* (B1, L* = 84.87 and 84.82, respectively). After 2 and 3 months of storage, significant differences were noted in the brightness of the cheeses. The decrease in cheese brightness during ripening is associated with the concentration of cheese components [49]. Furthermore, proteolysis that occurs during ripening can transform casein into a more soluble state and can cause a decrease in the L* parameter [50]. The decrease in L* value over time was associated with the partial oxidation during the storage period and the increase in microorganism counts in the samples [51].

Regarding the a* parameter, significant differences among the samples were observed after 2 and 3 months of storage. The color of the cheese R was redder (+a*) than B1 cheese (a* = 1.86–2.34) after 2 months of storage. Concerning the b* parameter, the yellow color changed over the storage period; after 1 month of storage, the studied cheeses were more yellow than after production (b* = 15.09–16.21, *p* < 0.05). Immediately after production and throughout storage, the R cheese was the most yellow. The increase in the a* and b*color parameters is mainly due to the increase in the concentration of the cheese components owing to dehydration throughout the ripening process [52]. It was thought that the increase in a* value was due to oxidation during storage or a possible increase in the number of microorganisms [53].

### 3.3. Microbiological Analysis

The tested cheeses were produced from raw milk. The microbiota from raw milk mainly affects the course of cheese maturation and cheese quality. Cheeses made from unpasteurized milk are very popular in markets worldwide. Their unique sensory attributes are desired by consumers. Some authors highlighted the health-promoting characteristics of specific environmental lactic acid bacteria as well as their impact on human health [54]. However, the consumption of raw milk cheeses can create a potential risk to human health [55,56,57,58,59]. To reduce the microbial risk associated with cheese made from raw milk, a longer period of ripening is required. On the other hand, milk pasteurization is not a guarantee of the microbiological safety of cheeses due to the possibility of secondary infections after production [60].

The results of the assessment of the microbiological quality of the studied acid-rennet cheeses are presented in Table 3. The number of LABs after production was similar, respectively, for B1 and R cheeses: 8.00 log CFU g^−1^ and 7.73 log CFU g^−1^. During the storage of B1 cheeses, a greater LAB number of about 2 logarithmic levels was found compared to the R cheeses. This proves that the starter culture, *L. brevis* B1, found good conditions for growth during the cheese production and maturation processes, as well as during storage, and predominates the microbiota of the final product. Therefore, it seems that the addition of *Levilactobacillus brevis* B1 can enhance the potential of natural bio-preservation of the cheese [61]. The *L. brevis* B1 strain was previously isolated from regional cheese, and its growth dynamics in the milk environment were found to be high [17], which seems to confirm that probably this strain caused the higher count of LAB in B1 cheese samples. However, in order to clearly confirm the presence of the B1 strain in cheese samples, an analysis at the genetic level should be performed in further studies.

The bacteria of the *Enterobacteriaceae* family in B1 and R cheeses after production were: 6.86 and 6.44 log CFU g^−1^, respectively (Table 3). After 3 months of storage, there was a decrease of *Enterobacteriaceae* in the B1 variant of studied cheeses (*p* < 0.05). The count of *Enterobacteriaceae* bacteria in the samples should be considered high, which is connected with health risks for consumers. *Enterobacteriaceae* belongs to the natural microbiota of milk. The activity of *Enterobacteriaceae* microorganisms can affect cheese ripening and influence the sensory characteristics of cheeses [62]. Their presence can indicate insufficient hygienic practices during cheesemaking [63,64]. However, the presence of *Enterobacteriaceae* may be due to the use of raw milk for production [65]. In the studied cheeses, there was no presence of the pathogenic bacteria *L. monocytogenes* and *Salmonella* in B1 and R cheeses after production and during storage. Assuming consumer safety is a crucial issue, the hygiene standards of cheesemaking and milking should be improved and continuously monitored [62]. In further studies, a deeper analysis of potential pathogenic bacteria should be performed.

In our study, a high number of yeasts and molds (approx. 4–5 log CFU g^−1^) were observed in cheeses with and without starter culture. LAB responsible for milk fermentation as well as starter cultures predominate the microbiota of rennet and ripened cheeses. Due to the production of substances such as bacteriocin, organic acids, and hydrogen peroxide, LAB can effectively inhibit the growth of pathogenic microorganisms that may be present in the complex microbiota of raw-milk cheeses [66].

### 3.4. Instrumental Texture Analysis

The results of the texture profile analysis of studied acid-rennet cheeses are shown in Table 4. The texture of cheeses depends on the quality of raw milk, technological processes, and biochemical transformations occurring during the production and maturation of cheeses [67].

The hardness 1 and 2 of the control (R) samples was higher than that of cheeses made using *L. brevis* (B1) after production and during 3 months of storage. The hardness of cheeses depends mainly on their water content [68,69]. The significantly (*p* < 0.05) higher hardness 1 and hardness 2 of R cheeses after production can be due to their lower water content (Table 1). The higher hardness 1 and hardness 2 after cheese production can be explained by the higher protein content of these cheeses [70,71]. A decrease in hardness 1 and hardness 2 of both R and B1 cheeses after 1 month of storage may be a consequence of proteolytic changes (resulting from bacterial activity) [72,73]. Our results were similar to the results of Aminifar and Emar Diome (2014), who found a reduction in the hardness of ovine and bovine soft cheeses (Lighvan cheeses) in the first month of ripening that did not change until the end of ripening [72]. Hardness 1 and 2 both B1 and R acid-rennet cheeses have been lowered during 1 month of storage, and show varying trends in the textural properties to the end of storage time. 

The hardness 2 of B1 acid-rennet cheese after production and during storage showed stable values, while changes in the hardness 2 of R cheese in subsequent study periods showed significant differences. The chewiness of R cheese after production was significantly higher than that of cheese with added *L. brevis* B1 strain (104.60 and 143.38 mJ for B1 and R cheeses, respectively) and decreased in both kinds of cheese during the storage period.

The gumminess of R cheese after production and during storage was higher than that of B1 cheese (Table 4); significant changes were recorded after production and 1 month of storage. The R cheeses after production and storage time showed higher cohesiveness than B1 cheese but were not statistically significant. Higher cohesiveness indicates that the cheese structure will not break up easily [74]. Harder cheeses are usually less cohesive compared with softer cheeses [75]. In our study, no such relationship was observed. Adhesiveness values varied from 0.73 mJ to 2.50 mJ for B1 and 0.70 mJ to 2.48 mJ for R. The adhesiveness of B1 and R cheeses was at a constant level, and there were no differences in the adhesiveness of the two cheese variants after production and during 3 months of storage.

### 3.5. Analysis of the Fatty Acid Composition, Cholesterol Content, and Lipid Quality Indices

The dominant fatty acids in the tested cheeses were saturated fatty acids (68.43–69.70%) and monounsaturated fatty acids (25.85–26.55%) (Table 5). The presence of polyunsaturated fatty acids was also found (2.85–3.18%). No significant differences were observed in the content of fatty acids during refrigerated storage. A significantly higher content of trans fatty acids was found in the variant of cheese B1 (1.80–1.85%) compared to cheese R (1.60–1.65%). The content of fatty acids in the n-3 group was 0.40% and in the n-6 group, it was from 1.95 to 2.30%. Significantly lower cholesterol content was observed for cheese B1 (24.68–29.05 mg/100 g of product) compared to cheese R (42.62–54.48 mg/100 g of product; *p* < 0.05). In the case of cheese B1, a statistically significant reduction in cholesterol content was recorded after 1 month (*p* < 0.05), while in the case of cheese R, a significant increase in the cholesterol content was observed after 3 months of storage. Some LAB strains can degrade food cholesterol. Some LAB species can reduce cholesterol levels in a food matrix. In the study of Fan et al. (2023), *Lactiplantibacillus plantarum* 54–1 was shown to be an efficient cholesterol-lowering strain in cheese [76]. Suggested mechanisms include direct transfer of cholesterol into the bacterial cytoplasm, incorporation into the cellular membrane, and binding to the surface of lactobacilli [77]. Another postulated mechanism is the conversion of cholesterol into coprostanol by LAB cholesterol reductase, which is active inside and outside bacterial cells [78]. In the study of Albano et al (2018), during 60 days of cheese ripening, a progressive reduction in cholesterol levels was observed, which is probably due to the fact that cholesterol is not released from LAB cells even in the mortality phase [79].

According to our data, it can be considered that *Levilactobacillus brevis* B1 has the potential to lower cholesterol content. However, it should be assessed and confirmed in further studies.

There were no significant differences in Lipid Quality Indices in the tested cheeses (Table 6). In general, the B1 cheese showed better indexes, including the Index of atherogenicity, Index of thrombogenicity, DFA, OFA, H/H, and HPI indexes, than the R cheese. Refrigerated storage did not significantly affect the changes in Lipid Quality Indices.

### 3.6. Sensory Evaluation

**Figure 1:**
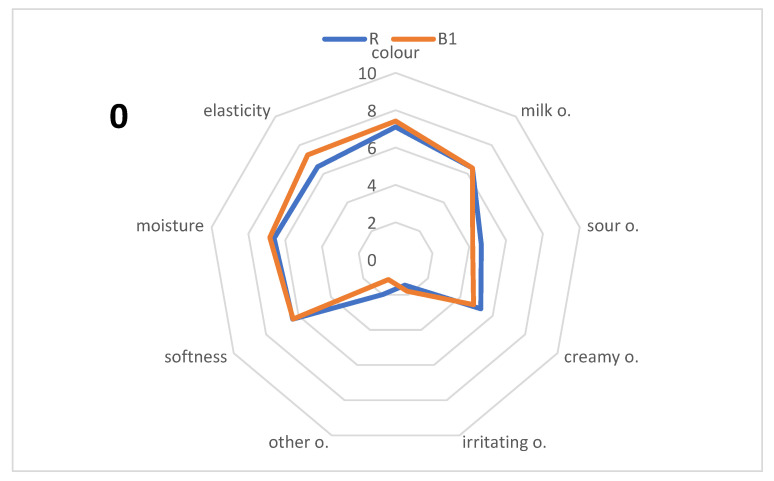

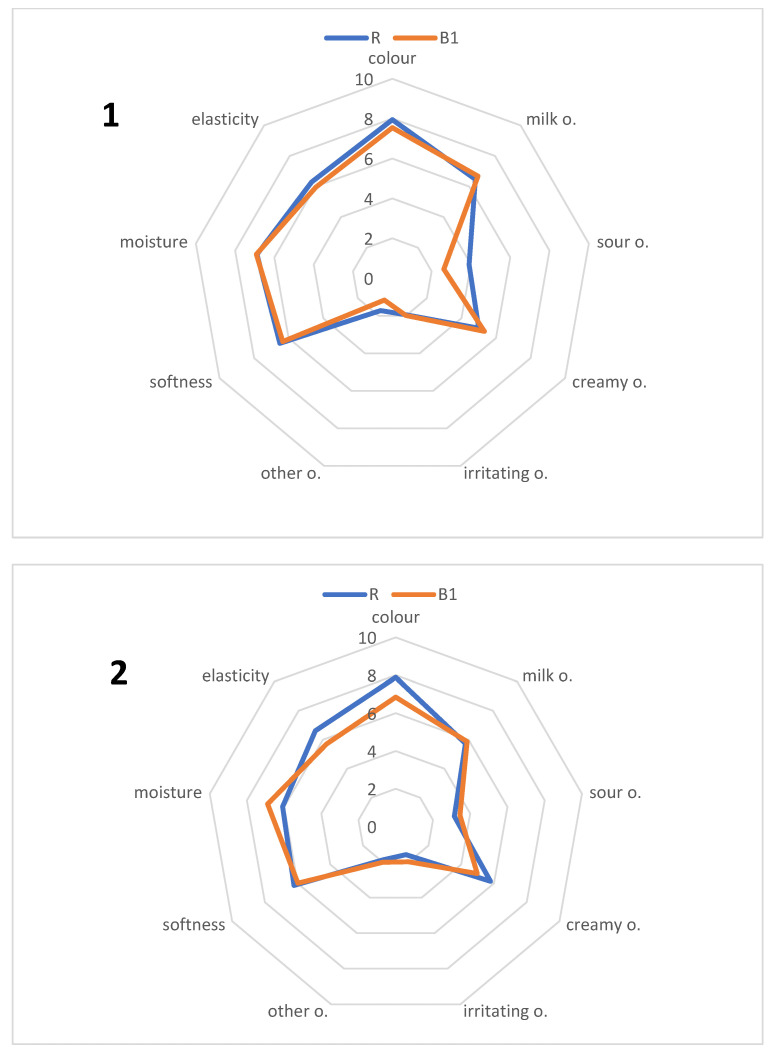

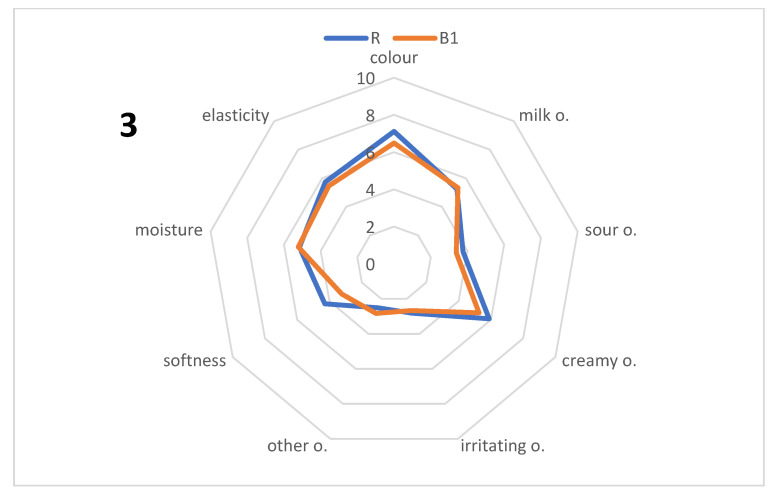
Sensory characteristics of cheeses after production and during storage (n = 16); 0—after production, 1—the first month of storage, 2—the second month of storage, and 3—the third month of storage; B1—acid-rennet cheese with *L. brevis* B1; R—acid-rennet cheese with buttermilk; o.—odor.

The analysis of the sensory quality (Figure 1) shows that during storage, insignificant variations in the perception of the color attribute of the cheeses during the entire 3-month storage period were observed. In the case of both types of acid-rennet cheeses, there was a decrease in the intensity of the sour note by about 1 unit, from 4.19 after production to 3.39 c.u. for B1 cheese, and from 4.65 to 3.76 c.u. for R cheese, was observed during 3 months (Figure 1). A decrease in the intensity of the milky odor of both types of cheeses during storage was also observed. This can be explained by the formation of new aroma attributes and, thus, by the weakening of those describing the product after production. Cheeses made from raw milk have intense lipolysis and proteolysis due to indigenous milk microorganisms, especially lactic acid bacteria. Lipid metabolism and the formation of free fatty acids, mainly short- and medium-chain are important in the process of ripening rennet cheeses and may determine the establishment of appropriate flavor and aroma compounds [80]. During the ripening of cheese also the proteolysis of peptides and free amino acids contributes to the cheese flavor [81,82]. Noteworthy is the increase in the intensity of “other” odor and odor attribute called “irritating” associated typically with the ripening process, which proves the changes in the maturation of cheeses taking place. During the 3 months of storage, a decrease in the softness, elasticity, and moisture of the B1 and R cheeses were observed. During storage, R cheese was rated as more yellow, but significant differences in the color between the two variants appeared after 2 and 3 months. A comparison of two studied samples B1 and R showed a similarity between both samples, which means that there is no statistically important impact of studied *L. brevis* B1 on sensory quality regarding the control sample (R) during 3 months of storage was observed.

## 4. Conclusions

In the present study, the possibility of using the novel starter culture has been evaluated. It was found that the number of LAB in acid-rennet cheeses with the addition of the *Levilactobacillus brevis* B1 strain was high after production and during storage (above 8 log CFU g^−1^), and in cheeses made with the *L. brevis* B1 strain, the number of LAB was higher by about 2 logarithmic orders compared to control cheeses. A high number of LAB bacteria can contribute to the microbiological stability of the product during storage. Due to the use of unpasteurized milk, special attention should be paid to the quality of the raw material and the hygienic conditions for the production and storage of cheeses.

The nutritional value of the cheeses was also affected by the addition of starter culture. The dominant fatty acids in the tested cheeses were saturated fatty acids and monounsaturated fatty acids. Significantly higher PUFA content during storage was observed for B1 cheeses. The B1 cheeses characterized lower cholesterol content compared to cheese R and showed better indexes, including Index of atherogenicity, Index of thrombogenicity, DFA, OFA, H/H, and HPI indexes than the R cheese. No effect of the tested *L. brevis* B1 on sensory quality was observed concerning the control cheeses during 3 months of storage.

The results of the research indicate the possibility of using the *Levilactobacillus brevis* B1 strain for the production of high-quality, potentially probiotic acid-rennet cheeses. Future research should be performed to prove the probiotic activity for human health of the acid-rennet cheese samples, as well as confirm the microbiological and toxicological safety of the novel product.

## 5. Patents

Patent No.: P.426002/ UP RP: Zielińska D., Ołdak A., Łepecka A., Kołożyn-Krajewska D. 2020: New strain of *Lactobacillus brevis* and application of a new strain of *Lactobacillus brevis bacteria.*

## Figures and Tables

**Table 1:** Chemical composition of acid-rennet cheeses after production.

Component	Variant of Cheese	Chemical Composition [%]
Water	B1	46.98 ± 0.27 ^b^
	R	42.88 ± 0.08 ^a^
Protein	B1	18.65 ± 0.05 ^a^
	R	19.90 ± 0.10 ^b^
Fat	B1	27.50 ± 0.20 ^a^
	R	30.03 ± 0.13 ^b^
NaCl	B1	1.40 ± 0.20 ^a^
	R	1.35 ± 0.05 ^a^
Phosphorus	B1	0.91 ± 0.01 ^a^
	R	0.95 ± 0.01 ^a^

Values shown are means ± SD, means in the column between B1 and R variants in the selected raw (specific component), with different lowercase letters (a,b) indicating significant difference (*p* < 0.05), B1—acid-rennet cheese with *L. brevis* B1; R—acid-rennet cheese with buttermilk, *n* = 3.

**Table 2:** Average values for pH, titratable acidity, oxidation-reduction potential, water activity, and color of tested cheeses after production and after 1, 2, and 3 months of storage.

Parameter	Variants of Cheeses	Time of Storage (Months)			
		0	1	2	3
pH	B1	5.13 ± 0.02 ^aB^	4.99 ± 0.02 ^aA^	4.92 ± 0.03 ^aA^	5.02 ± 0.05 ^aA^
	R	5.14 ± 0.01 ^aB^	5.03 ± 0.04 ^bA^	5.14 ± 0.03 ^bB^	5.19 ± 0.06 ^bB^
Titratable Acidity (SH°)	B1	225.50 ± 8.87 ^aC^	88.0 ± 2.45 ^aA^	91.0 ± 2.24 ^bA^	129.5 ± 9.10 ^bB^
	R	229.25 ± 5.54 ^aC^	83.5 ± 6.98 ^aA^	79.15 ± 1.84 ^aA^	96.5 ± 3.84 ^aB^
ORP (mV)	B1	398.05 ± 39.01 ^AB^	410.1 ± 4.78 ^aAB^	513.5 ± 4.32 ^aA^	491.9 ± 13.50 ^bA^
	R	479.65 ± 9.82 ^bAB^	392.83 ± 4.18 ^aA^	519.48 ± 1.15 ^aB^	442.68 ± 2.70 ^aAB^
a_w_	B1	0.995 ± 0.007 ^aA^	0.975 ± 0.004 ^aA^	0.960 ± 0.000 ^aA^	0.960 ± 0.000 ^aA^
	R	0.975 ± 0.007 ^bA^	0.976 ± 0.002 ^aA^	0.952 ± 0.005 ^bA^	0.947 ± 0.005 ^bA^
L*	B1	84.87 ± 1.37 ^aA^	84.82 ± 1.32 ^aA^	87.73 ± 1.00 ^bAC^	85.93 ± 1.24 ^bAB^
	R	85.03 ± 1.63 ^aAB^	84.22 ± 2.01 ^aA^	85.7 ± 1.83 ^aAB^	83.13 ± 2.35 ^aA^
a*	B1	1.43 ± 0.13 ^aA^	1.40 ± 0.20 ^aA^	2.34 ± 0.19 ^bC^	1.62 ± 0.11 ^bB^
	R	1.37 ± 0.17 ^aA^	1.34 ± 0.19 ^aA^	1.86 ± 0.50 ^aB^	1.43 ± 0.22 ^aA^
b*	B1	14.11 ± 0.80 ^aA^	15.09 ± 1.12 ^bA^	15.74 ± 1.11 ^aB^	15.32 ± 0.69 ^aB^
	R	14.93 ± 0.63 ^bA^	16.21 ± 0.98 ^bB^	17.02 ± 0.83 ^bC^	17.33 ± 0.51 ^bC^

Values shown are means ± SD, means in the same row with different uppercase letters (A–C) indicate significant difference (*p* < 0.05), means in the same column with different lowercase letters (a,b) indicate significant difference (*p* < 0.05), B1—acid-rennet cheese with *L. brevis* B1; R—acid-rennet cheese with buttermilk; time of storage (4 °C): 0- after production and after 1, 2, and 3 months of storage, n = 3.

**Table 3:** Microbiological analysis of tested cheeses.

Count of Microorganisms [log CFUg ^−1^]	Variant/Time of Storage	0	1	2	3
LAB	B1	8.00 ± 0.70 ^Aa^	9.06 ± 0.50 ^ABa^	9.09 ± 0.01 ^Ba^	9.09 ± 0.05 ^Ba^
	R	7.73 ± 0.21 ^Aa^	7.60 ± 0.02 ^Ab^	7.61 ± 0.01 ^Ab^	7.33 ± 0.26 ^Ab^
*Enterobacteriaceae*	B1	6.86 ± 0.11 ^Aa^	7.66 ± 0.20 ^Aa^	7.67 ± 0.19 ^Aa^	6.21 ± 0.13 ^ABa^
	R	6.44 ± 0.08 ^Aa^	6.98 ± 0.11 ^Aa^	6.44 ± 0.52 ^Ab^	6.32 ± 0.25 ^Aa^
Yeast and molds	B1	4.26 ± 0.51 ^Aa^	4.08 ± 0.18 ^Aa^	4.87 ± 0.12 ^Aa^	5.76 ± 0.08 ^ABa^
	R	3.96 ± 0.51 ^Aa^	3.98 ± 0.54 ^Aa^	5.37 ± 0.32 ^ABa^	4.65 ± 0.01 ^ABa^
*Salmonella* spp. in 25 g of product	B1	nd*	nd*	nd*	nd*
	R	nd*	nd*	nd*	nd*
*L. monocytogenes* in 25 g of product	B1	nd*	nd*	nd*	nd*
	R	nd*	nd*	nd*	nd*

Values shown are means ± SD, means in the same row with different uppercase letters (A,B) indicate a significant difference (*p* < 0.05), means in the same column with different lowercase letters (a,b) indicate a significant difference (*p* < 0.05), B1—acid-rennet cheese with *L. brevis* B1; R—acid-rennet cheese with buttermilk; nd*—not detected; time of storage (4 °C): 0- after production and after 1, 2, and 3 months of storage, *n* = 3.

**Table 4:** Texture analysis of acid-rennet cheeses after production and after 1, 2, and 3 months of storage.

Parameter	Variant/Time of Storage	0	1	2	3
Hardness Cycle 1 [N]	B1	26.13 ± 2.98 ^ABa^	19.67 ± 2.97 ^Aa^	20.58 ± 2.32 ^ABa^	28.17 ± 3.35 ^Ba^
	R	50.71 ± 13.95 ^Ab^	29.29 ± 4.26 ^Bb^	25.16 ± 3.72 ^Ba^	35.72 ± 2.81 ^Ca^
Adhesiveness [mJ]	B1	0.73 ± 0.44 ^Aa^	1.36 ± 0.72 ^Aa^	2.50 ± 0.51 ^Aa^	1.87 ± 0.42 ^Aa^
	R	0.70 ± 0.37 ^Aa^	2.07 ± 1.13 ^Aa^	1.50 ± 1.22 ^Aa^	2.48 ± 1.09 ^Aa^
Hardness Cycle 2 [N]	B1	16.25 ± 2.31 ^Aa^	13,35 ± 2.15 ^Aa^	13.59 ± 1.51 ^Aa^	18.64 ± 4.19 ^Aa^
	R	23.25 ± 8.27 ^Ab^	20.45 ± 3.50 ^Bb^	18.07 ± 2.73 ^Ba^	26.27 ± 1.77 ^Ab^
Cohesiveness	B1	0.46 ± 0.03 ^Aa^	0.30 ± 0.11 ^Ba^	0,18 ± 0.05 ^BCa^	0.20 ± 0.03 ^Ba^
	R	0.45 ± 0.11 ^Aa^	0.33 ± 0.05 ^ABa^	0.22 ± 0.02 ^Ba^	0.28 ± 0.07 ^Ba^
Springiness [mm]	B1	8.71 ± 0.16 ^Aa^	6.06 ± 1.09 ^Ba^	4.54 ± 0.73 ^Ca^	4.51 ± 0.78 ^Ca^
	R	8.68 ± 0.38 ^Aa^	6.24 ± 0.88 ^Ba^	4.48 ± 0.37 ^Ca^	5.06 ± 0.92 ^Ba^
Guminess [N]	B1	12.02 ± 1.70 ^Aa^	5.86 ± 2.25 ^Ba^	3.67 ± 0.86 ^Ba^	5.60 ± 1.41 ^Ba^
	R	16.63 ± 7.14 ^Ab^	9.47 ± 1.61 ^Bb^	5.63 ± 1.09 ^Ba^	10.01 ± 2.04 ^Ba^
Chewiness [mJ]	B1	104.60 ± 14.32 ^Aa^	37.20 ± 18.92 ^Ba^	17.00 ± 6.19 ^Ba^	25.13 ± 7.11 ^Ba^
	R	143.38 ± 54.91 ^Ab^	59.46 ± 15.27 ^Ba^	25.32 ± 5.64 ^Ca^	51.90 ± 19.77 ^Ba^

Values shown are means ± SD, means in the same row with different uppercase letters (A–C) indicate significant difference (*p* < 0.05), means in the same column with different lowercase letters (a,b) indicate significant difference (*p* < 0.05), B1—acid-rennet cheese with *L. brevis* B1; R—acid-rennet cheese with buttermilk; 0- after production and after 1, 2, and 3 months of storage.

**Table 5:** The content of fatty acids and cholesterol in the tested cheeses.

Parameter	Variant/Time of Storage	0	1	2	3
SFA [%]	B1	68.43 ± 0.05 ^Aa^	68.60 ± 0.00 ^Aa^	68.55 ± 0.15 ^Aa^	68.45 ± 0.00 ^Aa^
	R	68.83 ± 0.05 ^Aa^	69.70 ± 0.05 ^Aa^	68.95 ± 0.05 ^Aa^	68.85 ± 0.15 ^Aa^
MUFA [%]	B1	26.55 ± 0.05 ^Aa^	26.40 ± 0.00 ^Aa^	26.50 ± 0.00 ^Aa^	26.45 ± 0.10 ^Aa^
	R	26.48 ± 0.10 ^Aa^	25.85 ± 0.05 ^Aa^	26.40 ± 0.05 ^Aa^	26.35 ± 0.15 ^Aa^
PUFA [%]	B1	3.18 ± 0.05 ^Aa^	3.15 ± 0.05 ^Ab^	3.10 ± 0.05 ^Ab^	3.10 ± 0.00 ^Ab^
	R	3.08 ± 0.00 ^Aa^	2.85 ± 0.00 ^Aa^	2.95 ± 0.05 ^Aa^	2.90 ± 0.00 ^Aa^
trans [%]	B1	1.85 ± 0.05 ^Ab^	1.80 ± 0.00 ^Ab^	1.85 ± 0.00 ^Ab^	1.85 ± 0.05 ^Ab^
	R	1.62 ± 0.05 ^Aa^	1.60 ± 0.00 ^Aa^	1.65 ± 0.05 ^Aa^	1.65 ± 0.05 ^Aa^
n-3 [%]	B1	0.40 ± 0.00 ^Aa^	0.40 ± 0.00 ^Aa^	0.40 ± 0.00 ^Aa^	0.40 ± 0.00 ^Aa^
	R	0.40 ± 0.00 ^Aa^	0.40 ± 0.00 ^Aa^	0.40 ± 0.00 ^Aa^	0.40 ± 0.00 ^Aa^
n-6 [%]	B1	2.30 ± 0.00 ^Aa^	2.25 ± 0.05 ^Ab^	2.20 ± 0.05 ^Aa^	2.23 ± 0.01 ^Ab^
	R	2.10 ± 0.00 ^Aa^	1.95 ± 0.05 ^Aa^	2.05 ± 0.05 ^Aa^	2.00 ± 0.00 ^Aa^
Cholesterol [mg/100 g]	B1	29.05 ± 2.15 ^Ba^	24.68 ± 0.42 ^Aa^	25.35 ± 0.45 ^Aa^	25.00 ± 0.25 ^Aa^
	R	47.98 ± 1.00 ^Bb^	47.25 ± 2.05 ^Bb^	42.62 ± 0.65 ^Ab^	54.48 ± 1.80 ^Cb^

Values shown are means ± SD, means in the same row with different uppercase letters (A–C) indicate a significant difference (*p* < 0.05), means in the same column with different lowercase letters (a,b) indicate a significant difference (*p* < 0.05), B1—acid-rennet cheese with *L. brevis* B1; R—acid-rennet cheese with buttermilk; 0- after production and after 1, 2, and 3 months of storage; SFA—saturated fatty acids, MUFA—monounsaturated fatty acids, PUFA—polyunsaturated fatty acids, trans—trans fatty acids, n-3—fatty acids n-3, n-6—fatty acids n-6.

**Table 6:** The Lipid Quality Indices of the tested cheeses.

Parameter	Variant/Time of Storage	0	1	2	3
AI	B1	2.71 ± 0.00 ^Aa^	2.75 ± 0.00 ^Aa^	2.73 ± 0.00 ^Aa^	2.73 ± 0.00 ^Aa^
	R	2.76 ± 0.00 ^Aa^	2.78 ± 0.02 ^Aa^	2.78 ± 0.00 ^Aa^	2.79 ± 0.01 ^Aa^
TI	B1	2.08 ± 0.00 ^Aa^	2.11 ± 0.00 ^Aa^	2.09 ± 0.00 ^Aa^	2.09 ± 0.00 ^Aa^
	R	2.12 ± 0.00 ^Aa^	2.20 ± 0.02 ^Aa^	2.13 ± 0.00 ^Aa^	2.14 ± 0.00 ^Aa^
DFA	B1	40.73 ± 0.04 ^Aa^	40.55 ± 0.05 ^Aa^	40.30 ± 0.03 ^Aa^	40.40 ± 0.00 ^Aa^
	R	40.26 ± 0.02 ^Aa^	39.70 ± 0.01 ^Aa^	40.05 ± 0.01 ^Aa^	39.85 ± 0.06 ^Aa^
OFA	B1	46.70 ± 0.00 ^Aa^	47.15 ± 0.03 ^Aa^	46.75 ± 0.05 ^Aa^	46.80 ± 0.02 ^Aa^
	R	47.30 ± 0.05 ^Aa^	48.20 ± 0.01 ^Aa^	47.50 ± 0.05 ^Aa^	47.50 ± 0.00 ^Aa^
H/H	B1	0.50 ± 0.00 ^Aa^	0.49 ± 0.01 ^Aa^	0.49 ± 0.01 ^Aa^	0.49 ± 0.00 ^Aa^
	R	0.48 ± 0.00 ^Aa^	0.46 ± 0.01 ^Aa^	0.48 ± 0.02 ^Aa^	0.47 ± 0.02 ^Aa^
HPI	B1	0.38 ± 0.01 ^Aa^	0.37 ± 0.00 ^Aa^	0.37 ± 0.00 ^Aa^	0.37 ± 0.00 ^Aa^
	R	0.37 ± 0.00 ^Aa^	035 ± 0.00 ^Aa^	0.37 ± 0.00 ^Aa^	0.36 ± 0.00 ^Aa^

Values shown are means ± SD, means in the same row with different uppercase letters indicate a significant difference (*p* < 0.05), means in the same column with different lowercase letters indicate a significant difference (*p* < 0.05), B1—acid-rennet cheese with *L. brevis* B1; R—acid-rennet cheese with buttermilk; 0- after production and after 1, 2 and 3 months of storage; AI—Index of atherogenicity, TI—Index of thrombogenicity, DFA—Hypocholesterolemic fatty acids, OFA—Hypercholesterolemic fatty acids, H/H—The ratio of hypocholesterolemic and hypercholesterolemic fatty acids; HPI—Health Promoting Index.

## Data Availability

Data is contained within the article.
